# Supplementary material for: PgMYB1 Positively Regulates Anthocyanin Accumulation by Activating PgGSTF6 in Pomegranate
Source: Int J Mol Sci. 2023 Mar 28;24(7):6366. doi: 10.3390/ijms24076366 (PMC10094570; doi:10.3390/ijms24076366)
Supplement: Supplementary file 1 [file ijms-24-06366-s001.zip › Supplementary Material Figures.pdf]

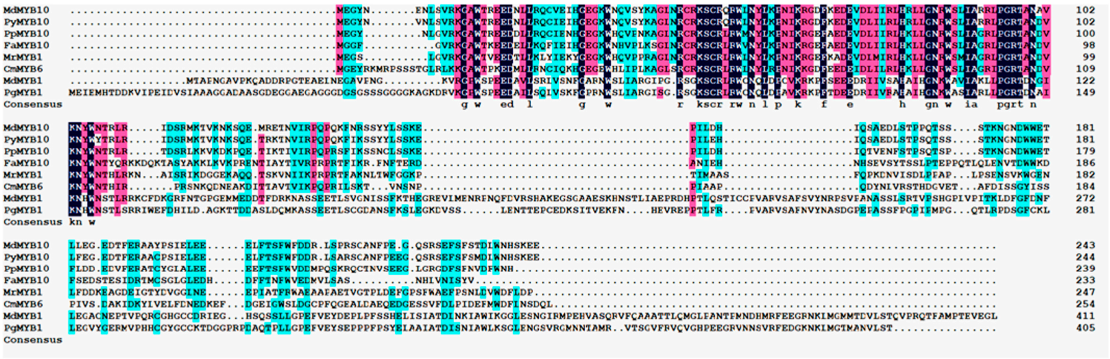

Figure S1 Comparison between amino acid sequences of PgMYB1 and their close homologs in plant species.

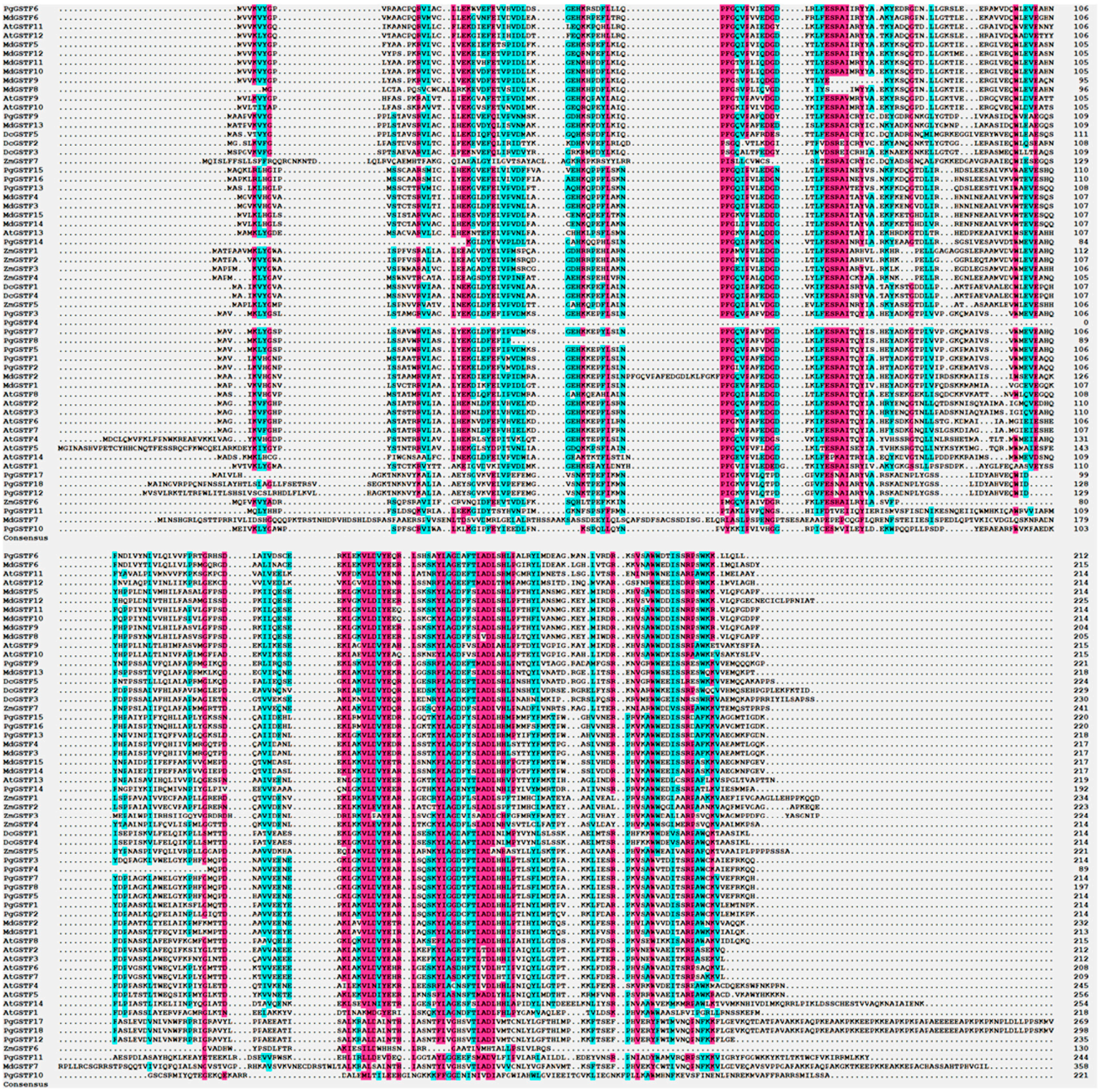

Figure S2 Comparison between PgGSTFs and their close homologs in plant species. Comparison of amino acid sequences of 18 PgGSTFs with their close homologs.

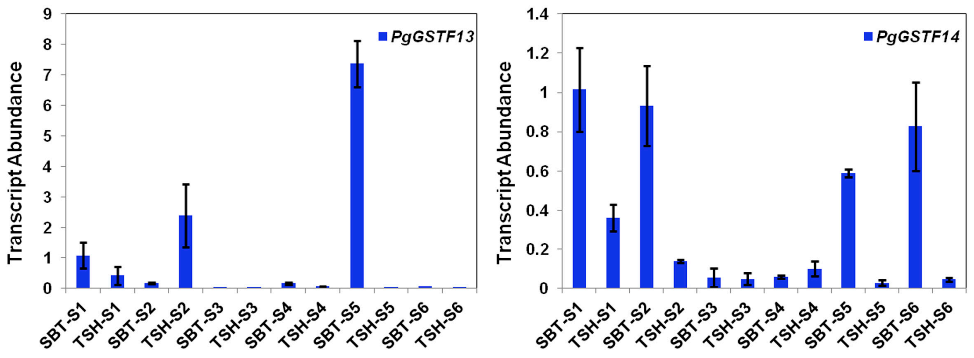

Figure S3 Expression pattern of *PgGSTF13* and *PgGSTF14* in different developmental stages of TSH and SBT. qRT-PCR analysis of *PgGSTF13* and *PgGSTF14* expression in different developmental stages of TSH and SBT. Error bars indicate ± SD (n=3, from three technical replicates). Values in Fig. S3 were derived from experiments that were performed at least three times with similar results, and representative data from one repetition were shown.

proPgGSTF6

AGGAGAAGATGACGAGGACGAGGAAGGATGAAAGGAACCTCTAAGAGCAGTGAATCGAGCAG
AAAGATCATCGCCTAGGACGGTGTTCAGCTCGTCGTCAACTTGAGGCGGAGGAGGTGAGGGT
TTTGGTCGTGAGAGGGATGGAGTCGGGGGAGGAGATTTGAGAGCTTGGAAGTAGCGAGAGA
GATCGATGATTGAGGGAATGAAAAGTAGTGCGGGCTACGTGGGAGTTGAAGGAAAGCTTCAA
AAGGACATCGTCATGTGCGGCCTGCAGAAGCTGTTCCACTTCATCCATACCTGAAGGTGGATT
TCCGAGGGTCGGAGGGGGAGGTAATTGAGCAAGCCCTGAAACTCCATCTCCAAAAGGGAAAT
GACGAGGCATGAGTAGTTGGGATGAGGGAAGGTTTCCTCCTTTTTCTGGAGAAAAACGGGAT
GAAGTAGGGGCTGGAAAGTAGCCGAGATTCACCGCAGGGGCAGATTGGAGAGAAGCTGAGC
GAGAAATGGAAGCTCGCATAAGGATAGGAAGAGGAAGAGGAAGACAAAGGAGAGGAAATTCA
TTTGAATTGGGACTGTGGGACGAAAGAGAAGGGGAAAGAGTGAGAGAATGAGAGAGAGAAG
GCATAGAAAGAAGATGATAATTTGAAGTTACATTAATTTGCCCAGCCATTACATCGATTCAGTCT
CTGAACAAATTAACGGAACAATAACGCTATCTGCCCCTTAGCCACTCAGACTAGATTGATGTAAC
CGGTGATTGATCTAATGTAACGCTACAACTCAAGGACTTGTGTTGCGAAAGTCGGGAACTAAAT
TGATGTAACCGAGCAAAGTCGAGGACCAATTTTATGTAACCGAAAAGTTATTGACGAAATTGAT
GCAAAAGTGCAAAGTCGGGGACGAAATTGATGCAAAATATAAGTCGGGAATGAAATTGCCTATT
TACTCATGTATGATTGAACAAGTGCGACCATTGTGCCTTTGAAATTAAATTGTGGAATATACAAAA
GGTCGATTGTTGGCTCGTACAAAGTTTTGTATGTAATGGATATATCAAGTGTCATTATGTGATAAG
ATATAACTAAAAACTATTTTTAACATGTCATAAATTTGTAATAACGCGTATTGATGTATTGAACACC
AATTAGTTTATGATACAAGTTGCATAATATAATCATTACATATAAGATTTTCTCGTTGGCTGAGGAT
TATATTTTTGTAAGTGTGCTTAATCAATGAGGAACGCAATATAGATTTTCCTTATTTCGATGCGTC
ACTACATGGAAAATTCCATAATGTAATCACTTTTTATTTGTGCAATTATTTTGAATCATAAAATTATA
ATTATTAATAAATAAATAAATC**CTGTTG**ATTATTCAACGATTAAAAAGCTGATTGCCCGTACAATTA
CTTGTAATATAGACCTTCCAATCACTGTACCCATTTAAAATTAATATTTTTGGAGAGCGGGGCAA
GCCAACCGCCGGTAGACGAGGCGCATGTGATAACCCCCGCGGATTGCTGCAACTAACTGCAA
CTACATGATTTTATTATGTAATTATTTTGCGCGGCTGTATATGCATCTGAATTGAGACTGGTCAGA
GAAGAGAGGCGTGCGCGCACTGACCCCACAGTCAAGTCCAGATAGTCAGTGAGTGTAATTTT
CTATAAATAAATAACAATCAATTAAAATACTTATAAATACCAATATATAATTTTATTGTGAGCGAATTT
CTGAATTTCTGAATTTGATAGGTACCCATTGACTGGTCCCGGTGAATTTCAACGGTTGTCGGTT
GGGTCTGGGTCGAGACAGATCTCACGTGGCAACCCCCACCCGTCGTAGACAAAATCACACTT
CAGTATACATATTTATATATATGTGTGTGTGTGTGTGTCTGTGCAGCAGAAATCAGATCCCCAACT
AGCCTGCACTGTCTTAGCTAGCTAGCTGCTGGGTGGGATATATTAAATTTCATCCAAAATTAAAA
GATCAGATCCCC

Figure S4 PgMYB1 may be able to bind to the *cis*-element of CTGTTG in the promoter region of *PgGSTF6*. Promoter sequence of *PgGSTF6.* PgMYB1 recognition site in promoter region of *PgGSTF6* is highlighted in bold with red color.
